# The contribution of preschool playground factors in explaining children's physical activity during recess

**DOI:** 10.1186/1479-5868-5-11

**Published:** 2008-02-26

**Authors:** Greet Cardon, Eveline Van Cauwenberghe, Valery Labarque, Leen Haerens, Ilse De Bourdeaudhuij

**Affiliations:** 1 Department of Movement and Sports Sciences, Ghent University, Watersportlaan 2, 9000 Ghent, Belgium; 2 European University College Brussels, Research Center for Education and Welfare, Campus Nieuwland, Nieuwland 168, 1000 Brussels, Belgium; 3 Research Foundation Flanders, Ghent University, Watersportlaan 2, 9000 Ghent, Belgium

## Abstract

**Background:**

Low levels of physical activity are characteristic in preschoolers. To effectively promote physical activity, it is necessary to understand factors that influence young children's physical activity. The present study aimed to investigate how physical activity levels are influenced by environmental factors during recess in preschool.

**Methods:**

Preschool playground observations and pedometry during recess were carried out in 39 randomly selected preschools (415 boys and 368 girls; 5.3 ± 0.4 years old). In order to examine the contribution of playground variables to physical activity levels, taking adjustment for clustering of subjects within preschools into account, multilevel analyses were conducted.

**Results:**

During recess boys took significantly more steps per minute than girls (65 ± 36 versus 54 ± 28 steps/min). In both genders higher step counts per minute were significantly associated with less children per m^2 ^and with shorter recess times. Only in boys a hard playground surface was a borderline significant predictor for higher physical activity levels. In girls higher step counts were associated with the presence of less supervising teachers. Playground markings, access to toys, the number of playing or aiming equipment pieces and the presence of vegetation or height differences were not significant physical activity predictors in both genders.

**Conclusion:**

In preschool children physical activity during outdoor play is associated with modifiable playground factors. Further study is recommended to evaluate if the provision of more play space, the promotion of continued activity by supervisors and the modification of playground characteristics can increase physical activity levels in preschoolers.

## Background

The childhood obesity epidemic is affecting even preschool children and reduced physical activity is an important contributor to this problem [1-4]. The National Association for Sport and Physical Education [5] suggests that preschool-aged children accumulate at least 120 minutes of physical activity per day, one-half of that time in structured physical activity and the remaining in unstructured free-play settings. However, according to the literature, preschoolers are characterized by low levels of physical activity and high levels of sedentary behaviour [6,7] and in previous research in 76 Flemish children attending preschool, it was shown that only 26% of the children accumulated at least 120 minutes of total physical activity per day [8]. Hence, there is an urgent need for effective interventions aimed at increasing physical activity in preschoolers. However, to effectively promote physical activity, it is necessary to understand the factors that influence physical activity in this young age group.

According to the recent review of Davison and Lawson [9], the role of supportive environment is important as a trigger of physical activity, particularly in children. However few studies, focusing on environmental correlates of physical activity, included preschool children. Furthermore these studies mainly focused on the home environment, like availability of home equipment and play spaces [10], and on local conditions, like neighbourhood safety [11,12].

Besides the home environment, the preschool environment may play an important role in achieving adequate physical activity levels for young children since in many countries most children spend extensive time in preschools. However, Pate et al [13], Finn et al [14] and Dowda et al [15] reported low levels of physical activity during preschool attendance. The latter two studies [14,15] also reported significant differences in physical activity levels between children in different preschools and advocated the provision of sufficient space for the children, teacher-training and the organisation of physical activities at preschool. Furthermore, Boldemann et al [16] found in a sample of 197 4- to 6-year olds that spacious preschool environments with trees, shrubbery, and broken ground triggered physical activity.

In most preschool programs, break times with unstructured free play are scheduled for more periods each day, making it an important environmental factor for the promotion of physical activity. While the terminology of break time at pre-school may differ across countries, in the present study the term "recess" is used. Recess is typically held outdoors and allows children to move freely. However it was shown that 4- to 5-year-old children spent the majority of recess break time in sedentary activities [17]. In the literature, different opportunities, like playground redesign, paintings of court markings, fun trails and hopscotches [18-20], provision of game equipment [21], and teacher supervision [22], have been evaluated in the scope of activity engagement at recess in elementary school children. However it is unclear which playground factors correlate with physical activity during recess in preschool environments. If policies are to be designed and disseminated for the purpose of increasing physical activity among preschool children, then those policies should be developed on the basis of an improved understanding of basic aspects of physical activity in early childhood. Hence the main purpose of the present study is to determine which environmental factors contribute to physical activity levels during recess in preschool boys and girls. Additionally gender differences in physical activity levels during recess will be explored.

## Methods

### Subjects

The study was executed in Flanders, the Dutch speaking part of Belgium, located in the centre of Europe. In Flanders almost all elementary schools have a public preschool program (2213 schools with a preschool program; 95%), which allows children to participate from the age of 2.5 years old. The programs are free and virtually all children attend. Since they are organized in the elementary school settings, large and safe indoor and outdoor spaces to play are available for most preschool programs. Moreover, all preschool programs are lead by college educated teachers. A random sample of 45 preschools from 40 different municipalities in Flanders was asked to participate in the study. A sample of 40 schools agreed to participate. All parents (829) of the 4- and 5-year-old children of the 40 participating schools were informed about the study by an information letter. The evaluations were considered to be part of the psychological, medical and social counselling provided by the school, for which all parents signed a consent form. The study was approved by the Ethical committee of the Institutional Review Board at Ghent University.

One school was excluded due to rainy weather on the three days of measurements attempts. Data for 27 children were omitted due to measurement errors or unrealistic data (< 15 steps recorded), possibly due to resetting. The final sample consisted of 415 boys and 368 girls (see table 1) from 39 preschools (15 to 30 children from each school). Measurements were performed between October 2006 and February 2007, which is winter time in Belgium. The average day temperature during data collection was 7 degrees Celsius.

**Table 1:** Descriptive characteristics and steps per minute of the sample by sex.

	**Boys **N= 415	**Girls **N= 368
Age (years)	5.2 (± 0.4)	5.3 (± 0.4)
Length (m)	1.14 (± 0.05)	1.13 (± 0.05)
Weight(KG)	20.6 (± 3.5)	20.2 (± 3.2)
Steps per minute	65 (± 36)	54 (± 28)
Steps per minute square root	7.7 (± 2.1)	7.1 (± 1.9)

### Measures and procedure

#### Physical activity levels

Step counts were assessed using the Yamax Digi-walker pedometer TYPE SW-200 (Yamax corp, Japan), which is an unobtrusive instrument measuring 19 mm × 39 mm × 52 mm that uses a horizontal spring-suspended mechanical lever arm to measure vertical movement. Pedometry has been recommended due to children's intermittent pattern of physical activity [23] and findings of McKee et al [24] supported the utility of the Digiwalker pedometer for assessing physical activity in young children. They reported a strong relationship between the Children's Activity Rating Scale and step counts per 3 minutes in 30 3- to 4-year-olds while undertaking normal school activities during a 1-hr period in a nursery setting. Furthermore Eisenmann and Wickel [25] compared the cost of locomotion between a 6-year-old and a 12-year-old and concluded that the number of steps taken is equivalent among humans of varying body size if taken at the same speed. Moreover a previous study showed good correlation with accelerometer data (r = 0.73) and good receptivity of a pedometer in preschool children [26]. Pedometers were attached at the waist, above the right hip. To minimize reactivity, the pedometers were attached to the children and the children were familiarized with the instrument upon arrival at school, thus 90 to 120 minutes before registration. Pedometers were reset to zero when leaving the building for recess and step counts were registered when re-entering after recess. Steps per minute were calculated, making use of a stop watch to measure recess durations. The stop watch was started when 50 % of the children had entered the playground and stopped when 50 % had left the playground. To avoid losing recess time, pedometers were not sealed, but the children were told not to open the pedometers. Moreover it was observed in a previous study that many children have difficulty opening the pedometer [26]. Registrations were only performed when it was not raining during recess, thus when the weather permitted outdoor play.

#### Playground factors

Factors of the playground environment served as independent variables. All playground features were recorded by members of the research team, who visited the schools. The presence or absence of the following playground factors were recorded: markings, soft surface (partly soft surfaces were also coded as "present"), vegetation, height differences, and availability of toys for a minimum of 10% of the children, The numbers of aiming equipments (e.g. goals, poles with one or more baskets), the numbers of playing equipment (e.g. swings, slides, climbing racks) and the numbers of teachers supervising during the PA registrations were counted by the researchers. The researchers measured all playgrounds to determine the play space per child. Playground features that were not accessible for the preschoolers during the measurements, were not included (e.g. none accessible field due to wet grass). Additionally, all playgrounds were photographed for verification by the first author.

### Statistical analyses

Preliminary analyses consisted of descriptive statistics of sample characteristics using SPSS for windows (12.0). Univariate regression analyses were conducted using MLwiN version 2.02. As the dependent variable was skewed to the right, the square root was taken to improve the normality of this variable. To investigate the Univariate relationships between gender and step counts, taking into account adjustment for clustering of subjects within preschools, a single-predictor two-level model (pupil-school) was used. Because most studies in children showed gender differences in types of physical activity correlates [9,27] analyses examining the contribution of playground variables to physical activity levels were conducted in boys and girls separately. To investigate the Univariate relationships between step counts and each of the independent variables (number of children per m^2^, number of supervising teachers, recess duration, number of playing equipments, number of aiming equipment pieces, presence of a soft surface, markings, height differences, vegetation, and the access to toys) a single-predictor two-level (school-pupil) model was used. To test the significance of the variance at the school level Z-scores were calculated. Intra-school correlation was calculated to assess a measure of similarity between the same pupils in each of the schools. Intra-school correlations measure the extent to which step counts of pupils in one school resemble to each other as compared to those from pupils in different schools; it gives a measure of the percentage of variance in step counts that may be attributed to differences between schools. The alpha level was set at 0.05 for all analyses.

## Results

The average number of children per m^2 ^was 0.15 (± 0.08, range 0.02–0.44). The average recess duration was 24.27 minutes (± 11.05, range 9–50). The mean number of aiming equipment pieces on the playground was 1.79 (± 1.2, range 0–4) and schools had on average 2.53 (± 2.15, range 0–9) pieces of playing equipment on the playground. Markings were present on 24 of the 39 playgrounds, 20 playgrounds had height differences, the playground surface was partly soft in 20 schools, in 23 schools toys were available for minimum 10 % of the children and in 21 preschools vegetation was present on the playground.

Variance at the school level was borderline significant among boys (*Z *= 3.4, *p *< 0.07) and girls (*Z *= 3.6, *p *= 0.06). Among boys, 27% of the variance in step counts was attributed to the differences between schools, among girls 35% of the variance in step counts was attributed to the differences between schools. Being a girl as compared to a boy was associated with significantly lower activity levels (β = -0.622, SE = 0.280, *p *≤ 0.05). In table 2 Univariate multi-level analyses of the associations between playground factors and step counts per minute (square root transformed) can be found. Among boys step counts were significantly predicted by number of children per m^2 ^(β = -4.635, SE = 2.104, *p *≤ 0.05) and recess duration (β = -0.001, SE = 0.000, *p *≤ 0.001). The presence of a soft playground surface was a borderline significant predictor (β = -0.687, SE = 0.369, *p *≤ 0.07). Among girls, step counts were significantly predicted by number of children per m^2 ^(β = -5.411, SE = 2.163, *p *≤ 0.01), number of supervising teachers (β = -0.526, SE = 0.239, *p *≤ 0.05) and recess duration (β = -0.001, SE = 0.000, *p *≤ 0.001). Lower numbers of children per m^2 ^and shorter recesses were related to increased step counts per minute in both sexes. The presence of hard surfaces was related to higher activity levels among boys. Less supervising teachers on the playground was related to higher activity levels among girls. The number of playing equipments, number of aiming equipment pieces, presence markings, height differences, vegetation and the access to toys were not significantly related to step counts in both genders. Non-transformed data analyses gave identical results, except for a harder playground surface, which was significantly associated with higher step counts in boys (*p *≤ 0.05).

**Table 2:** Univariate multi-level analyses of the associations between playground factors and step counts per minute (square root transformed).

**Factors**	**Univariate multi-level analyses β (SE)**	
	
	**Boys**	**Girls**
**Children/m^2^**	- 4.635 (2.104)*	- 5.411 (2.163)**
**Supervising teachers**	- 0.347 (0.235)	- 0.526 (0.239)*
**Aiming equipment**	0.106(0.152)	0.010 (0.016)
**Playing equipment**	- 0.056 (0.084)	- 0.255 (0.418)
**Recess duration**	- 0.001 (0.000)****	- 0.001 (0.000)***
**Ground Surface**	- 0.687 (0.369)(*)	- 0.601 (0.392)
**Markings**	0.613 (0.384)	0.424 (0.412)
**Vegetation**	- 0.044(0.390)	- 0.386 (0.403)
**Height differences**	0.614 (0.380)	0.459 (0.401)
**Toys**	- 0.150 (0.392)	- 0.035 (0.089)

(*) p ≤ 0.07, *: p ≤ 0.05, ** p ≤ 0.001, ***:p ≤ 0.001

## Discussion

In the present study in preschools, average step count values per minute were 65 in boys and 54 in girls. The higher activity levels in boys are in line with the literature [6,7,13,14,17,28] and builds on to the evidence that lower levels of PA in girls compared to boys are already significant at young age.

The step count values of the present study were considerably higher than the average step counts reported by Boldemann et al [16], in 4- to 6 year olds, during preschool attendance. In the latter study boys took on average 21 steps per minute and girls took 18 steps per minute. But these data were not specific for recess time and also included structured sedentary activities, like having lunch. The present average step count values approximate taking only one step per second. Apparently during recess the engagement in vigorous physical activity was limited and possibly, as found by Mc Kenzie et al [17], large parts of recess times may have been spent sedentary. Hence there are opportunities to increase activity levels at recess in preschoolers. Furthermore it was found in the present study in boys and in girls respectively, that 27 % to 35 % of variance in step counts may be attributed to differences between schools.

In both genders more space per child was found to be associated with more physical activity during recess. Therefore preschools should be encouraged to provide sufficient space for recesses, if necessary by splitting into groups with different recess times. Furthermore it was observed that boys and girls took fewer steps per minute when recesses lasted longer. This may be due to the possibility that children show a burst of activity when they first go outside which subsides with time. In the present study recess times varied from 9 minutes to 50 minutes. Possibly physical activity levels decreased after a certain amount of time, due to fatigue or getting bored. Also McKenzie et al [17] found in preschoolers that activity levels declined as recess time elapsed. Efforts to increase children's outdoor play time need to be advocated, since children are presumably still more active overall if they are outdoors for longer periods of time. However, efforts to promote continued activity during outdoor play may be needed or more recess periods per day may be preferable.

An interesting finding of the present study is the fact that children were less active when more teachers were supervising. However this was only significant in girls. This can be explained by the fact that many teachers supervise sitting down or standing still. Since many children, and presumably especially girls, prefer to stay close to the teachers, more supervising teachers may cause decreased activity levels. Consequently efforts seem useful to inform and encourage present and future preschool teachers to promote activity during recess (e.g. by playing with the children or at least encourage active play). Incorporating physical activity promotion in the training of future preschool teachers may enable them to implement the principles in their daily work and to enter into a professional career with a positive attitude toward physical activity promotion. According to the findings of Boldemann et al [8] in environments with trees, shrubbery and broken ground step counts/min were higher than in delimited environments with little vegetation. In the present study height differences and vegetation were present in about half of the schools but the presence was not significantly associated with higher step counts. This can be explained by the fact that the extend of the height differences and vegetation on the playgrounds was only limited. On the other hand a harder ground surface was a borderline significant predictor for higher step counts in boys only. A possible explanation is that the spontaneous behaviour may differ between both sexes, with boys being more triggered by harder ground surfaces, which are mainly used for more sports-related, competitive activities.

A remarkable finding of the present study is the fact that the availability of toys, the presence of aiming or playing equipment, like swings or slides, and the presence of markings was not associated with more physical activity. Possibly the choice of toys (e.g. hoops), equipment pieces (e.g. swing) or markings (mainly field markings) were not optimal in the observed preschools and results may be different when focusing on certain types of toys, equipment or markings. Another explanation may be that toys and equipment often lead to standing in line to use the piece of toy or equipment. In the study of Zask et al [22] equipment availability was also not significant PA predictor in elementary school children, except for balls. Further study is needed to evaluate if specific toys, equipments or markings may be more successful to trigger physical activity and to evaluate if triggering may appear when they are available for all children.

A first limitation of the present study is that all data were collected during winter. Therefore physical activity levels possibly suffered from seasonal influence. However Belgium has a mild climate, measurements were only taken when the weather permitted outdoor playing and according to the findings of Fisher et al [29] seasonality plays only a limited role in physical activity in young children.

A second limitation is the use of pedometers, which may not capture or underestimate some activities among young children, like swinging or crawling. Strengths of the present study are the relatively large sample size, the use of an objective physical activity measure and observation of the playground environment, and the use of multilevel analyses to take into account adjustment for clustering of subjects within preschools.

## Conclusion

The present study contributed to the dearth of literature focusing on the correlates of physical activity in preschool children. It can be concluded from the present study that in preschool children physical activity during recess is associated with modifiable playground factors. Since many children attend preschool, there is a great potential to increase activity levels in preschoolers. Studying the effects of intervening on these factors is of interest. Meanwhile it seems plausible to recommend preschools to provide sufficient play space, to encourage supervisors to promote activity during recess, and to organize several recess periods during the day.

## Competing interests

The author(s) declare that they have no competing interests.

## Authors' contributions

GC, VL and IDB conceived the study and contributed to the planning and the design of the study. GC, VL and EVC collected the data and conducted data manipulation and analyses. LH contributed to the statistical analyses. GC wrote the manuscript. VL, IDB and EVC supplied comments. All authors read and approved the final manuscript.
